# Identifying gaps in HIV service delivery across the diagnosis-to-treatment cascade: findings from health facility surveys in six sub-Saharan countries

**DOI:** 10.7448/IAS.20.1.21188

**Published:** 2017-01-12

**Authors:** Kathryn Church, Kazuyo Machiyama, Jim Todd, Brian Njamwea, Mary Mwangome, Vicky Hosegood, Janet Michel, Samuel Oti, Constance Nyamukapa, Amelia Crampin, Nyaguara Amek, Gertrude Nakigozi, Denna Michael, F Xavier Gómez-Olivé, Jessica Nakiyingi-Miiro, Basia Zaba, Alison Wringe

**Affiliations:** ^a^ Department of Population Health, London School of Hygiene & Tropical Medicine, London, UK; ^b^ African Population and Health Research Center, Nairobi, Kenya; ^c^ Ifakara Health Institute, Ifakara, United Republic of Tanzania; ^d^ Department of Social Statistics & Demography, University of Southampton, Southampton, UK; ^e^ Africa Centre for Population Health, Mtubatuba, South Africa; ^f^ Manicaland Centre for Public Health Research, Harare, Zimbabwe; ^g^ Malawi Epidemiology and Intervention Research Unit, Lilongwe, UK; ^h^ Kenya Medical Research Institute and the Centers for Disease Control, Kisumu, Kenya; ^i^ Rakai Health Sciences Program, Uganda Virus Research Institute, Rakai, Uganda; ^j^ Tazama Project, Tanzania National Institute for Medical Research, Mwanza, Tanzania; ^k^ MRC/Wits Rural Public Health and Health Transitions Research Unit (Agincourt), School of Public Health, University of the Witwatersrand, Johannesburg, South Africa; ^l^ MRC/UVRI Uganda Research Unit on AIDS, Entebbe, Uganda

**Keywords:** HIV, ART, PMTCT, retention, health services, facility surveys, multi-country, continuum

## Abstract

**Introduction:** Despite the rollout of antiretroviral therapy (ART), challenges remain in ensuring timely access to care and treatment for people living with HIV. As part of a multi-country study to investigate HIV mortality, we conducted health facility surveys within 10 health and demographic surveillance system sites across six countries in Eastern and Southern Africa to investigate clinic-level factors influencing (i) use of HIV testing services, (ii) use of HIV care and treatment and (iii) patient retention on ART.

**Methods:** Health facilities (*n* = 156) were sampled within 10 surveillance sites: Nairobi and Kisumu (Kenya), Karonga (Malawi), Agincourt and uMkhanyakude (South Africa), Ifakara and Kisesa (Tanzania), Kyamulibwa and Rakai (Uganda) and Manicaland (Zimbabwe). Structured questionnaires were administered to in-charge staff members of HIV testing, prevention of mother-to-child transmission (PMTCT) and ART units within the facilities. Forty-one indicators influencing uptake and patient retention along the continuum of HIV care were compared across sites using descriptive statistics.

**Results:** The number of facilities surveyed ranged from six in Malawi to 36 in Zimbabwe. Eighty percent were government-run; 73% were lower-level facilities and 17% were district/referral hospitals. Client load varied widely, from less than one up to 65 HIV testing clients per provider per week. Most facilities (>80%) delivered services or interventions that would support patient retention in care such as delivering free services, offering PMTCT within antenatal care, pre-ART monitoring and adherence counselling. Many facilities under-delivered in several areas, however, such as targeted testing for high-risk groups (21%) and mobile testing (36%). There were also intra-site and inter-site differences, including in the delivery of Option B+ (ranging from 6% in Kisumu to 93% in Kyamulibwa), and nurse-led ART initiation (ranging from 50% in Kisesa to 100% in Karonga and Agincourt). Only facilities in Malawi did not require additional lab tests for ART initiation. Stock-outs of HIV test kits and antiretroviral drugs were particularly common in Tanzania.

**Conclusions:** We identified a high standard of health facility performance in delivering strategies that may support progression through the continuum of HIV care. HIV testing policy and practice was particularly weak. Inter- and intra-country differences in quality and coverage represent opportunities to improve the delivery of comprehensive services to people living with HIV.

## Introduction

In 2015 in Eastern and Southern Africa, 10.3 million people were accessing antiretroviral therapy (ART), representing an estimated 54% [50–58%] of all people living with HIV (PLHIV) in the region [1]. Ambitious HIV programmes have resulted in substantial declines in mortality rates among HIV-infected adults, estimated at 58% since the initial expansion of ART in a recent community level cohort analysis in six countries in Eastern and Southern Africa [2].

A large body of evidence, however, indicates that services are inadequately promoting access to testing or progression onto treatment for those diagnosed positive. A 2012 systematic review and meta-analysis in sub-Saharan Africa found that, on average, 39% of PLHIV were tested and knew their status; 57% of those diagnosed positive underwent assessment of ART eligibility and fewer (51%) returned for the result; among the newly diagnosed, 41–64% were found to be eligible for ART (depending on initiation criteria), among whom only 66% started ART; among those not yet eligible, a median of 45% remained in pre-ART care [3]. Another systematic review of 39 patient cohorts in the region found average retention on ART of 65% at three years [4]. Differences in progression through this “cascade” between or within countries are likely to impact on country-level variations in mortality among PLHIV.

**Figure 1.**
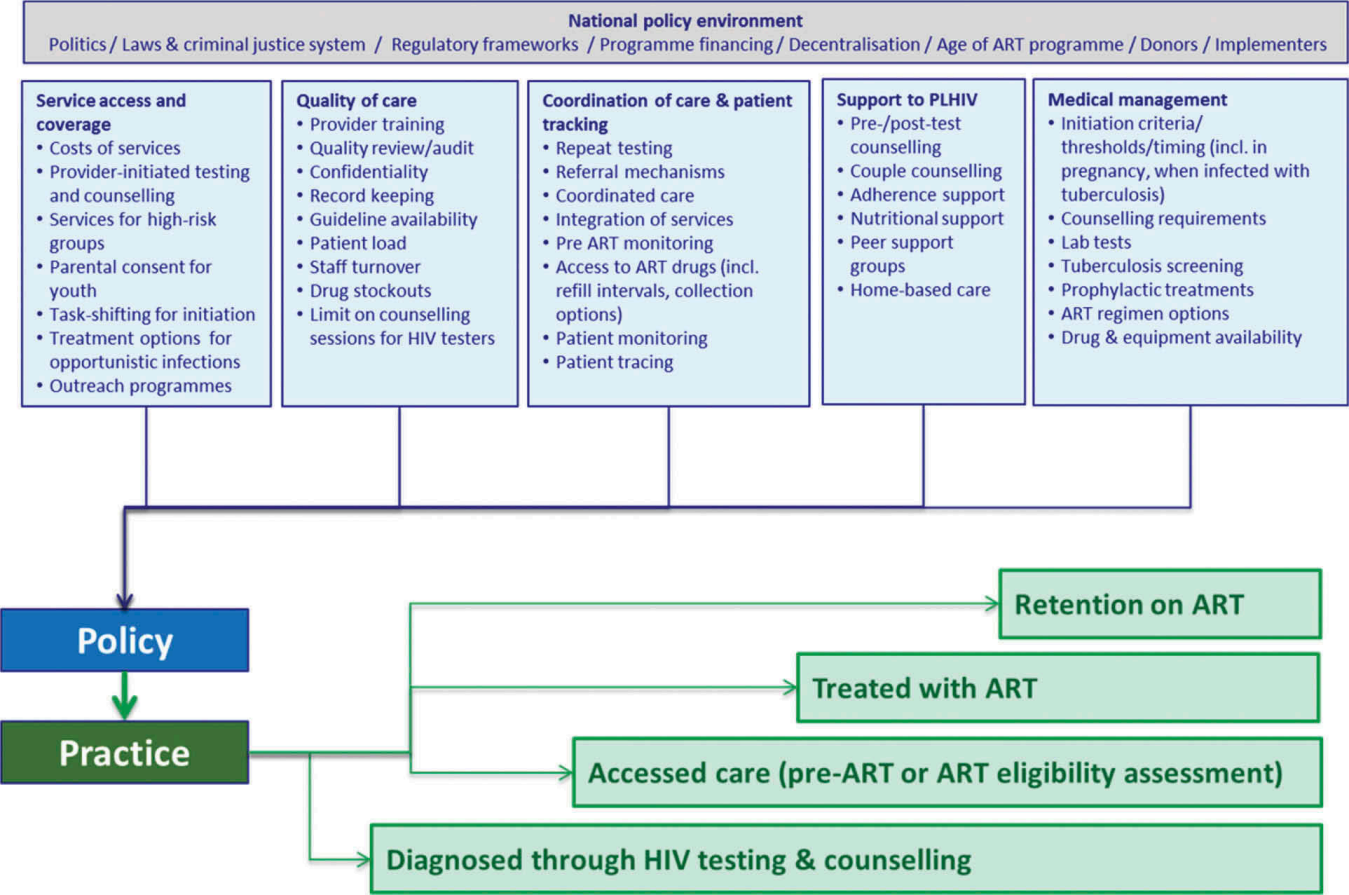
Conceptual framework identifying health system factors influencing access to adult HIV services through the cascade.

While engagement of PLHIV with services and subsequent progression through the cascade may stem from broader socio-economic, cultural or political influences, HIV service coverage and quality play a critical role [5]. Several systematic reviews have identified a range of health service influences on engagement of PLHIV with care, including attributes of service access and coverage; the quality of service provision; the coordination of care and follow-up; support given to PLHIV; and the clinical management of patients [6–9]. We subsequently incorporated these factors into a conceptual framework (Figure 1) to guide a broader study of policy and programmatic influences on cascade progression and adult HIV-related mortality in sub-Saharan Africa, conducted through the network for Analysing Longitudinal Population HIV/AIDS data in Africa (ALPHA, http://alpha.lshtm.ac.uk/).

We previously used this framework to review national adult HIV policies across the six sub-Saharan countries with generalized HIV epidemics where ALPHA collects mortality data [10]. We found that while policies were consistent in many areas (e.g. guarantees of free HIV services, promotion of opt-out HIV testing, regular pre-ART CD4 monitoring, task-shifting for ART initiation and integrated service promotion), there were also wide variations (e.g. targeted testing of high-risk groups, referral to peer support or home-based care, requirements of laboratory testing before ART initiation). The variation was surprising since many national programmes often follow standardized WHO recommendations [10].

Here we build on the policy review by investigating and comparing health facility practices that may influence access of PLHIV to adult HIV services within the 10 health and demographic surveillance system (HDSS) sites in Eastern and Southern Africa participating in the larger ALPHA mortality study: Kisumu (KEMRI/CDC) and Nairobi (Korogocho and Viwandani settlements) (both Kenya); Karonga (Malawi); Agincourt and uMkhanyakude (South Africa); Ifakara and Kisesa (Tanzania); Kyamulibwa and Rakai (Uganda); and Manicaland (Zimbabwe). As countries in the region adopt UNAIDS “90-90-90” targets and “test and test” policies, it is important to identify areas of strength and weakness in HIV service provision across the continuum of care.

## Methods

### Study settings

**Figure 2.**
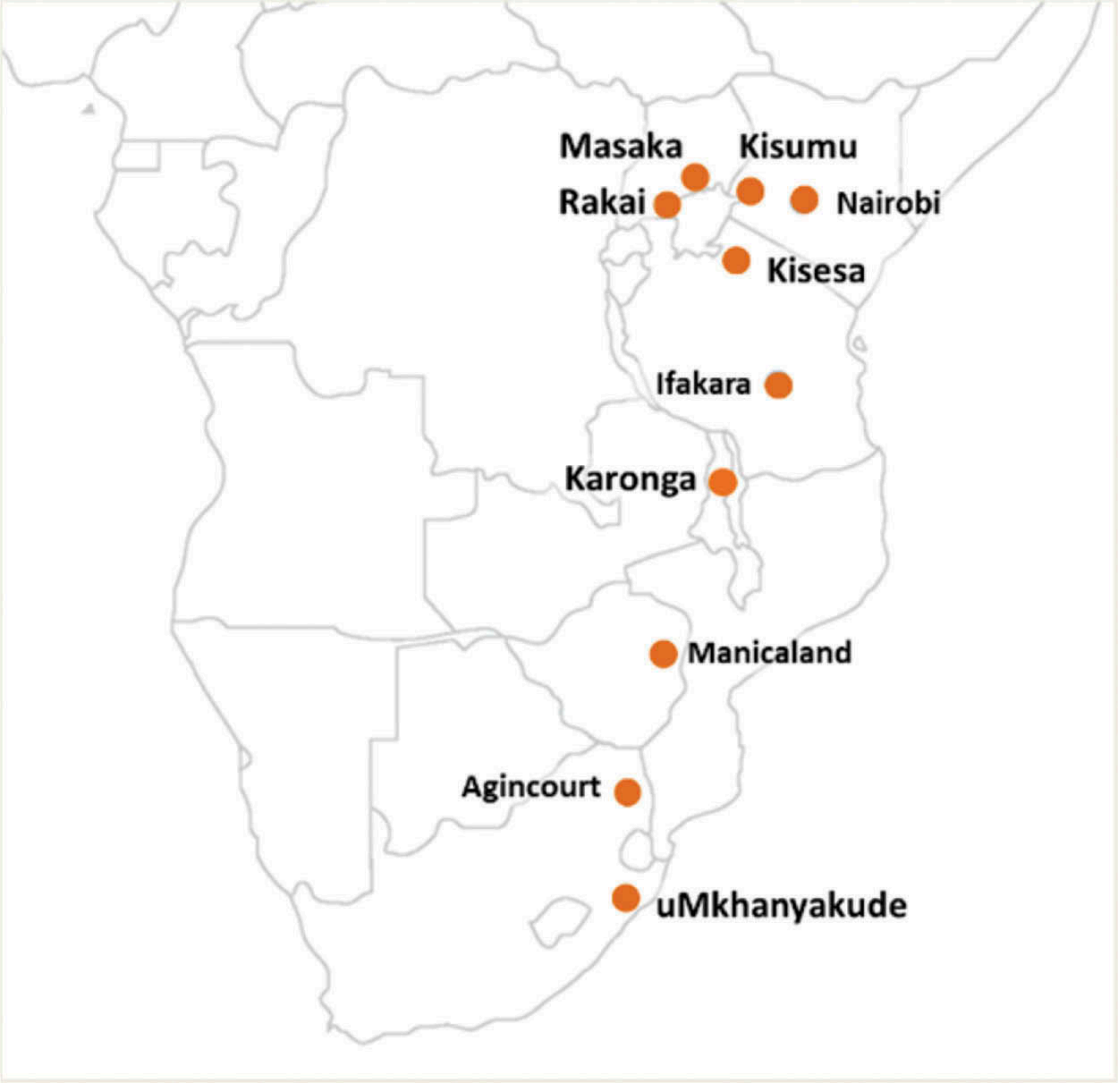
Location of the ALPHA Network member study sites.

Study sites were located in Eastern and Southern Africa (Figure 2), and key characteristics are highlighted in Table 1. More detailed information has been provided by Reniers et al. [2].

**Table 1.** Study setting and sampling information for the facility surveys, by site

Country	HDSS site	Size of HDSS site (km^2^)	Population of HDSS site	HIV prevalence	No. of facilities surveyed/total no. facilities in HDSS*	No. facilities outside HDSS site surveyed*	Sampling strategy
Kenya	Nairobi	Korogocho: 0.97 km^2^ Viwandani: 0.5 km^2^	72,557	12%	10/0	10	No HIV facilities in HDSS. Convenience sample of different types of HIV facilities used by residents: five on edge of HDSS, the remainder 2–15 km away
	Kisumu (KEMRI/CDC)	369 km^2^	141,956	15%	34/34	0	All facilities
Malawi	Karonga	135 km^2^	39,045	7%	6/7	1	All facilities, except one small private clinic
South Africa	Agincourt	420 km^2^	90,000	19%	9/10	2	All facilities, except one public–private health centre
	uMkhanyakude	438 km^2^	90,000	33%	17/7	10	All facilities, along with 10 facilities in the wider district also supported by the HDSS management
Tanzania	Ifkakara	2400 km^2^	169,000	7%	12/19	7	All facilities with ≥100 patients per month, including seven outside the HDSS used by residents
	Kisesa	150 km^2^	30,486	7%	8/5	3	All facilities, including three referral hospitals outside HDSS site
Uganda	Rakai	320 km^2^	32,109	13%	14/17	0	All facilities supported by Rakai Health Sciences Program
	Kyamulibwa	54.3 km^2^	21,450	9%	9/3	6	All facilities, including facilities outside HDSS used by residents
Zimbabwe	Manicaland	36,459 km^2^	11,139	15%	36/95	2	All main hospitals (five) plus random sample of clinics used by HDSS residents, plus two facilities outside HDSS used by residents

* *Facilities on the border with or outside the HDSS site were included if they were commonly used by residents of the sites.*

### Sampling of health facilities within sites

Health facilities providing HIV services to their HDSS population were surveyed, including government-, NGO-, and privately-run facilities (including inside, on the border of, or just outside surveillance areas). All facilities provided HIV testing and counselling (HTC), most provided prevention of mother-to-child transmission (PMTCT) and/or ART services. In some, a sample of facilities was surveyed: in Nairobi, a convenience sample was taken to include different types or levels of health facility used by HDSS residents; in Ifakara, only facilities with a patient load ≥100/month were surveyed (Table 1).

### Data collection, processing and analysis

The questionnaire was developed by reviewing existing HIV health facility surveys, including the World Health Organization Service Availability and Readiness Assessment surveys, and an instrument from Manicaland developed by Imperial College London [11]. Indicator selection was based on the conceptual framework shown in Figure 1.

The questionnaire covered health facility characteristics and staffing, and HTC, PMTCT and ART service provision practices. These included indicators related to the five themes in the conceptual framework and the policy review: (i) service access and coverage; (ii) quality of care; (iii) coordination of care and patient tracking; (iv) support to PLHIV; and (v) medical management.

The questionnaire was administered in English to the staff manager at each facility or within relevant sub-units. Interviewers observed the availability of treatment guidelines and consulted pharmacy records for patient numbers, drug stocks and test availability. Data collection took place between July 2013 and January 2015. Data from eight sites were entered centrally into the MS SQL Server (Microsoft Corp). Data were cleaned, merged and exported for analysis using Stata 12 (Stata Corp). Two sites, Kisumu and Manicaland, entered their own data which were subsequently merged into the pooled data set. Missing data are indicated in the tables and denominators adjusted.

For each site, service provision is presented using descriptive statistics. For continuous data, the median and range from facilities within each site are shown; for categorical variables, the number and proportion of facilities are shown. Since most sites included all facilities in operation, no formal statistical comparisons between sites have been made. Bar charts were produced to examine the distribution of a selection of key indicators of interest within the four service areas across the 10 sites.

### Ethics

Ethical approval was received from each site from a local regulatory authority, and from the London School of Hygiene and Tropical Medicine ethics committee (no. 8891-1). The ALPHA network data sharing agreement covered data sharing between sites.

## Results

**Figure 3.**
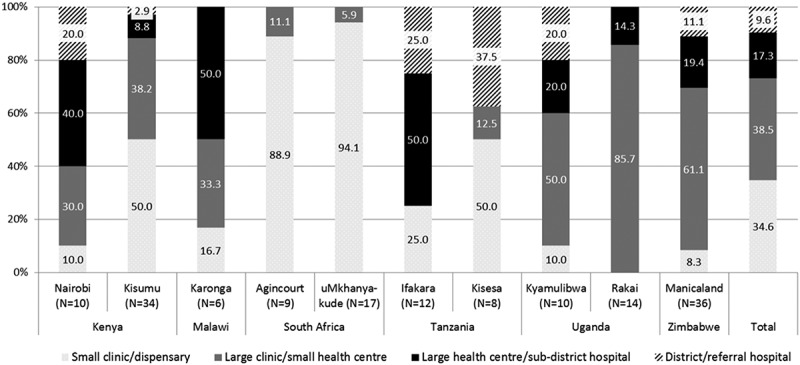
Type of facilities surveyed, by site*. *Small clinic/dispensary: see only outpatients. Large clinic/small health centre: have limited no of beds (for maternal deliveries) and may be headed by clinical officer/medical officer. Large health centre/sub-district hospital: have capacity for inpatients. Referral hospital: district, provincial or national hospital receiving referrals from smaller hospitals.

One hundred and fifty-six health facilities in the 10 sites participated in the analysis, ranging from six in Karonga to 36 in Manicaland (Table 2). Health facilities surveyed were either large health centres or hospitals (27%) or smaller health centres, clinics or dispensaries (73%) (Figure 3). South African sites had the highest proportion of small clinics (89% in Agincourt, 94% in UmKhanyakude).

**Table 2.** Clinic overview, by site

	**Kenya**				**Malawi**		**South Africa**				**Tanzania**				**Uganda**				**Zimbabwe**			
	**Nairobi**		**Kisumu**		**Karonga**		**Agincourt**		**uMkhanyakude**		**Ifakara**		**Kisesa**		**Kyamulibwa**		**Rakai**		**Manicaland**		**Total (%)**	
**Total clinics(*****n*****(%))**	**10**	**(100.0)**	**34**	**(100.0)**	**6**	**(100.0)**	**9**	**(100.0)**	**17**	**(100.0)**	**12**	**(100.0)**	**8**	**(100.0)**	**10**	**(100.0)**	**14**	**(100.0)**	**36**	**(100.0)**	**156**	**(100.0)**
**Total clinics offering ART (*****n*****(%))**	**8**	**(100.0)**	**32**	**(100.0)**	**5**	**(100.0)**	**9**	**(100.0)**	**17**	**(100.0)**	**12**	**(100.0)**	**4**	**(100.0)**	**10**	**(100.0)**	**14**	**(100.0)**	**15**	**(100.0)**	**126**	**(100.0)**
***Management authority (n******(%))***																						
Government	6	(60.0)	30	(88.2)	3	(50.0)	9	(100.0)	17	(100.0)	10	(83.3)	6	(75.0)	4	(40.0)	12	(85.7)	27	(75.0)	124	(79.5)
Faith-based org.	3	(30.0)	3	(8.8)	2	(33.3)	0	(0.0)	0	(0.0)	1	(8.3)	1	(12.5)	3	(30.0)	0	(0.0)	7	(19.4)	20	(12.8)
Other NGO	1	(10.0)	0	(0.0)	1	(16.7)	0	(0.0)	0	(0.0)	1	(8.3)	0	(0.0)	3	(30.0)	2	(14.3)	0	(0.0)	8	(5.1)
Private-for-profit	0	(0.0)	1	(2.9)	0	(0.0)	0	(0.0)	0	(0.0)	0	(0.0)	1	(12.5)	0	(0.0)	0	(0.0)	2	(5.6)	4	(2.6)
***HIV*-*****related services (n(%))***																						
HIV testing	10	(100.0)	34	(100.0)	6	(100.0)	9	(100.0)	17	(100.0)	12	(100.0)	8	(100.0)	10	(100.0)	14	(100.0)	36	(100.0)	156	(100.0)
PMTCT	10	(100.0)	34	(100.0)	5	(83.3)	9	(100.0)	17	(100.0)	12	(100.0)	7	(87.5)	8	(80.0)	14	(100.0)	35	(97.2)	151	(96.8)
HIV care (incl. pre-ART)	8	(80.0)	32	(94.1)	5	(83.3)	9	(100.0)	17	(100.0)	12	(100.0)	4	(50.0)	10	(100.0)	14	(100.0)	36	(100.0)	147	(94.2)
HIV treatment	8	(100.0)	32	(100.0)	5	(100.0)	9	(100.0)	17	(100.0)	12	(100.0)	4	(100.0)	10	(100.0)	14	(100.0)	15	(45.5)	126	(87.5)
Lab services	6	(60.0)	19	(55.9)	0	(0.0)	8	(88.9)	3	(17.6)	8	(66.7)	4	(50.0)	10	(100.0)	14	(100.0)	8	(22.2)	80	(51.3)
***Human******resourcesandpatient load (median, range)***																						
No. of clinicians^†^	2.5*	(0.0–8.0)	1.0	(0.0–34.5)	0.5	(0.0–2.5)	0.0	(0.0–0.5)	0.5	(0.0–0.5)	0.8*	(0.0–9.5)	2.0	(1.0–10.0)	3.0	(2.0–6.5)	2.0	(0.0–7.0)	0.0	(0.0–6.0)	0.8	(0.0–34.5)
No. nurses/midwives	6.5*	(0.0–18.0)	2.0	(0.0–62.0)	1.5	(0.0–8.0)	5.0	(4.0–10.0)	5.0	(2.0–19.0)	2.5*	(0.0–9.0)	1.0	(0.0–13.0)	4.0	(0.0–9.0)	3.3	(1.0–8.0)	1.0	(0.0–128.0)	3.0	(0.0–128.0)
No. counsellors	2*	(0.0–13.0)	0.3	(0.0–7.0)	3.3	(2.0–4.5)	2.0	(2.0–4.0)	2.5	(1.0–6.0)	0*	(0.0–1.0)	2.0	(0.0-9.0)	1.5	(0.0–67.5)	1.0	(0.0–10.0)	0.0	(0.0–5.0)	1.0	(0.0–67.5)
No. HIV testing clients/wk	78*	(11–1237)	39*	(0–206)	20	(9–30)	46*	(25–105)	38*	(5–221)	16**	(4–24)	30	(1–505)	19*	(1–2011)	58	(4–262)	11*	(2–45)	25.0	(0–2011)
No. of weekly HIV testing clients/staff^‡^	1.3*	(0.8–42.0)	3.7*	(0.0–41.2)	1.3	(0.7–10.0)	5.4*	(2.5–6.4)	2.6*	(0.7–10.1)	2.7**	(0.5–6.9)	4.2	(0.2–64.8)	1.8*	(0.0–4.3)	6.9	(0.5–29.3)	2.1*	(0.3–7.7)	2.9	(0.0–64.8)
Staff turnover^§^	10.5	(0.0–86.7)	0	(0.0–170.0)	22.5	(6.7–45.5)	12.5	(0.0–40.0)	66.7	(0.0–125.0)	0	(0.0–37.5)	5	(0.0–37.5)	1.5	(0.0–20.0)	16	(0.0–50.0)	0	(0.0–100.0)	1	(0.0–170.0)
***Human resources for ART (among clinics with ART on site)***																						
No. ART clients/wk	50**	(35–141)	37**	(0–154)	3	(1–4)	21**	(10–226)	8	(1–1477)	34**	(13–51)	228**	(5–451)	3	(0–270)	49	(19–508)	11**	(1–49)	28	(0–1477)
No. of weekly ART clients/clinician or nurse	4.8**	(3.3–8.8)	11.7**	(0.0–38.5)	1	(0.1–3.2)	2.9**	(1.6–45.2)	1.9	(0.2–75.7)	6.4**	(2.3–15.6)	10.6**	(1.6–19.6)	0.3	(0.0–29.8)	11	(4.8–253.9)	4.1**	(0.8–45.9)	6.1	(0.0–253.9)

*At least one site with missing data, **>10% of sites with missing data; denominators may vary for categorical variables. ^†^Doctor, clinical officer, assistant medical officer. ^‡^Nurse, midwife, nursing aide, counsellor or community outreach worker. ^§^No. staff left in past year as percentage of total staff (nurses, clinicians, aides, counsellors, outreach); figures over 100% indicate more staff left than are currently employed.

### Clinic overview and staffing

Facility characteristics are presented in Table 2. Most facilities were government-run (80%). All provided HIV testing, 97% provided PMTCT services, 94% provided HIV care, 88% provided ART and 52% provided laboratory services. Client flow varied by clinic type (data not shown) and site: median HIV testing clients averaged <20 per week in Ifakara, Kyamulibwa and Manicaland, but reached 78 in Nairobi. There was less variation in client load between sites (weekly HTC clients per provider ranging between 1.3 in Nairobi and Karonga to 6.9 in Rakai) than within sites (ranging between 0.2 and 65 HTC clients per week in Kisesa; and 5–254 ART clients per week in Rakai). Annual staff turnover varied widely from 0% in Kisumu, Ifakara and Manicaland to 67% in uMkhanyakude.

### Influences on access to HIV testing

**Figure 4.**
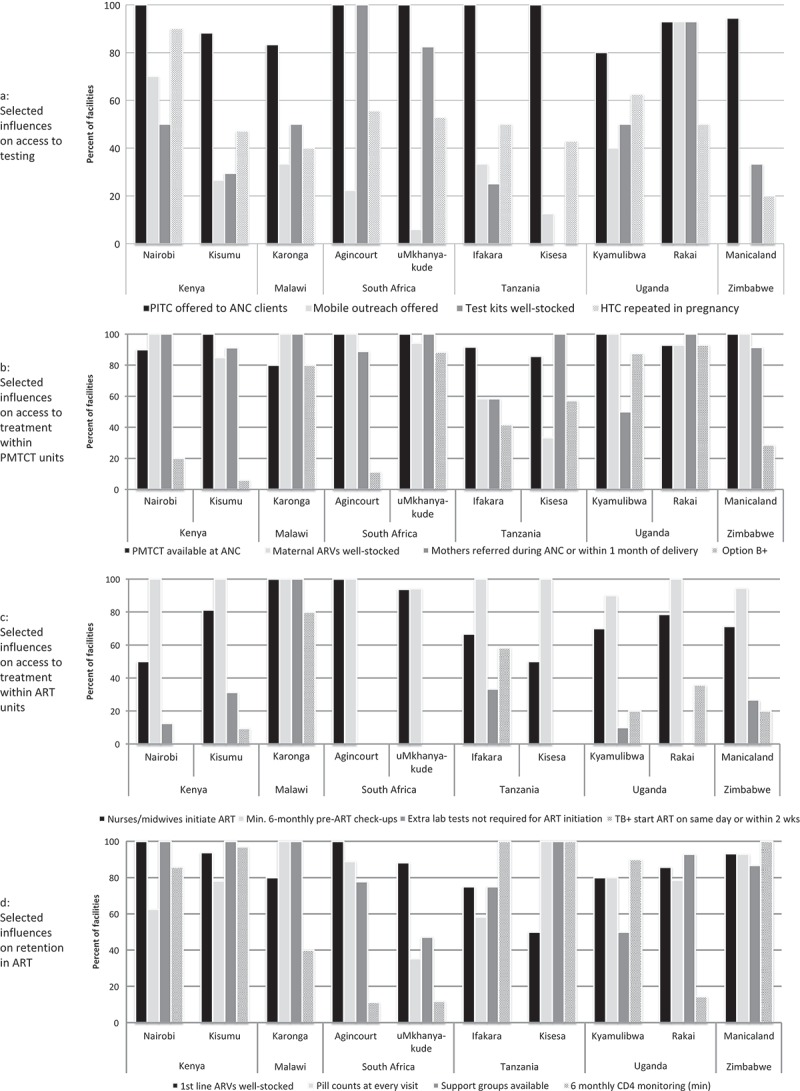
Selected factors influencing access to HIV services.

Influences on access to HIV testing are presented in Table 3 and Figure 4a. HTC was freely available in most facilities, but provider-initiated testing and counselling (PITC) in ante-natal care (ANC) was not universal in Kisumu, Karonga, Kyamulibwa, Rakai and Manicaland. Very few facilities explicitly offered HTC to high-risk groups and most did not offer mobile outreach.

**Table 3.** Influences on access to HIV testing

	Kenya				Malawi		South Africa				Tanzania				Uganda				Zimbabwe			
	Nairobi		Kisumu		Karonga		Agincourt		uMkhanya-kude		Ifakara		Kisesa		Kyamulibwa		Rakai		Manicaland		Total	(%)
**Total no. clinics (*n* (%))**	**10**	**(100.0)**	**34**	**(100.0)**	**6**	**(100.0)**	**9**	**(100.0)**	**17**	**(100.0)**	**12**	**(100.0)**	**8**	**(100.0)**	**10**	**(100.0)**	**14**	**(100.0)**	**36**	**(100.0)**	**156**	**(100.0)**
***Service access******andcoverage (n(%))***																						
Free HTC	9	(90.0)	34	(100.0)	6	(100.0)	9	(100.0)	17	(100.0)	12	(100.0)	7	(87.5)	8	(80.0)	14	(100.0)	31	(86.1)	147	(94.2)
PITC offered to ANC clients	10	(100.0)	30	(88.2)	5	(83.3)	9	(100.0)	17	(100.0)	12	(100.0)	8	(100.0)	8	(80.0)	13	(92.9)	34	(94.4)	146	(93.6)
HTC to high-risk groups (sex workers, MSM, drug users)	7	(70.0)	9	(26.5)	0	(0.0)	0	(0.0)	14	(82.4)	0	(0.0)	2	(25.0)	0	(0.0)	1	(7.1)	0	(0.0)	33	(21.2)
Mobile outreach offered	7	(70.0)	9	(26.5)	2	(33.3)	2	(22.2)	1	(5.9)	4	(33.3)	1	(12.5)	4	(40.0)	13	(92.9)	**		43	(35.8)
***Quality of care (n******(%))***																						
National testing guidelines available^†^	9	(90.0)	22	(64.7)	6	(100.0)	9	(100.0)	17	(100.0)	11	(91.7)	5	(62.5)	5	(50.0)	7	(50.0)	26**	(100.0)	117	(75.0)
At least one staff received training on HIV testing in past two years	8	(80.0)	31	(91.2)	6	(100.0)	7	(77.8)	12	(70.6)	7	(58.3)	6	(75.0)	4	(40.0)	13	(92.9)	22	(61.1)	116	(74.4)
HTC providers counsel max 15 clients per day	7	(70.0)	17	(50.0)	3	(50.0)	0	(0.0)	3	(17.6)	0	(0.0)	4	(50.0)	4	(40.0)	3	(21.4)	**		41	(26.8)
QOC audits at least once/yr	10	(100.0)	32	(94.1)	6	(100.0)	8	(88.9)	17	(100.0)	11	(91.7)	6	(75.0)	10	(100.0)	11*	(84.6)	32*	(91.4)	143	(92.9)
Test kits well-stocked^‡^	5	(50.0)	10	(29.4)	3	(50.0)	9	(100.0)	14	(82.4)	3	(25.0)	0	(0.0)	5	(50.0)	13	(92.9)	12	(33.3)	74	(47.4)
**Coor*****dination of care and patient tracking (n(%))***																						
Repeat test advised after window period	8	(80.0)	28	(82.4)	3	(50.0)	8	(88.9)	17	(100.0)	12	(100.0)	7	(87.5)	10	(100.0)	14	(100.0)	35	(97.2)	142	(91.0)
Testing repeated three months after first test in pregnancy and/or in third trimester^§^	9	(90.0)	16	(47.1)	2**	(40.0)	5	(55.6)	9	(52.9)	6	(50.0)	3**	(42.9)	5**	(62.5)	6**	(50.0)	7*	(20.0)	68	(45.6)
Check if HIV+ registered in care	8	(80.0)	33	(97.1)	3	(60.0)	8	(88.9)	16	(94.1)	8	(66.7)	5	(62.5)	9	(90.0)	14	(100.0)	32*	(94.1)	136	(88.9)
***Support to PLHIV (n******(%))***																						
Pre-test counselling always provided	10	(100.0)	31	(91.2)	5	(83.3)	9	(100.0)	17	(100.0)	11	(91.7)	2**	(28.6)	8	(80.0)	9	(64.3)	35	(97.2)	137	(88.4)
Individual as well as group pre-test counselling is offered	10	(100.0)	34	(100.0)	6	(100.0)	8	(88.9)	17	(100.0)	11	(91.7)	8	(100.0)	9	(90.0)	10	(71.4)	36	(100.0)	149	(95.5)
Post-test counselling always provided	9	(90.0)	33	(97.1)	6	(100.0)	9	(100.0)	17	(100.0)	8*	(72.7)	4	(50.0)	10	(100.0)	14	(100.0)	35	(97.2)	145	(93.5)

*At least one site with missing data, **>10% of sites with missing data; denominators may vary for categorical variables. ^†^Seen or not seen, any guideline. ^‡^Stock-outs ≤1 time in past year, or having no stock outs lasting for two weeks or more. ^§^In clinics with ANC only.

Quality of care factors influencing HTC included availability of national guidelines, found in all facilities in Karonga, Agincourt, uMkhanyakude and Manicaland; regular staff training, which was only conducted in all facilities in Malawi; and regular quality audits, which were conducted nearly everywhere. Stock-outs of testing kits occurred most rarely in Agincourt and Rakai. Repeat testing after the window period was commonly conducted, but less so during pregnancy. Most facilities (89%) checked whether PLHIV ultimately registered in care, although this was lower in Karonga (60%).

Provision of pre-test counselling was generally common, although only universal in Nairobi, Agincourt and uMhkanyakude, and infrequent in Kisesa (29%).

### Influences on access to HIV treatment in PMTCT

Factors influencing access to HIV treatment were investigated in PMTCT units (*n* = 151) (Table 4 and Figure 4b). Free PMTCT services were provided everywhere except Nairobi (70%), Kyamulibwa (75%) and Manicaland (43%). Most facilities had well-stocked maternal PMTCT drugs (average 89%), but only 58% of Ifakara and 33% of Kisesa facilities were well-stocked.

**Table 4.** Influences on access to HIV treatment within PMTCT units

	Kenya				Malawi		South Africa				Tanzania				Uganda				Zimbabwe			
	Nairobi		Kisumu		Karonga		Agincourt		uMkhanya-kude		Ifakara		Kisesa		Kyamulibwa		Rakai		Manicaland		Total	(%)
**Total no. clinics offering PMTCT (*n* (%))**	**10**	**(100.0)**	**34**	**(100.0)**	**5**	**(100.0)**	**9**	**(100.0)**	**17**	**(100.0)**	**12**	**(100.0)**	**7**	**(100.0)**	**8**	**(100.0)**	**14**	**(100.0)**	**35**	**(100.0)**	**151**	**(100.0)**
***Service access and******coverage (n(%))***																						
Free PMTCT	7	(70.0)	34	(100.0)	5	(100.0)	9	(100.0)	17	(100.0)	12	(100.0)	7	(100.0)	6	(75.0)	14	(100.0)	15	(42.9)	126	(83.4)
PMTCT available at ANC†	9	(90.0)	34	(100.0)	4	(80.0)	9	(100.0)	17	(100.0)	11	(91.7)	6	(85.7)	8	(100.0)	13	(92.9)	35	(100.0)	146	(96.7)
***Quality of care (n******(%))***																						
Maternal ARVs well-stocked^‡^	9*	(100.0)	28*	(84.8)	5	(100.0)	9	(100.0)	16	(94.1)	7	(58.3)	2**	(33.3)	8	(100.0)	13*	(92.9)	35	(100.0)	132	(89.2)
***Coor*******dination of care and patient tracking (n*(%))***																						
HIV Tx given on same day as ANC services	6	(60.0)	29	(85.3)	5	(100.0)	9	(100.0)	16	(94.1)	7	(58.3)	6	(85.7)	7	(87.5)	13	(92.9)	35	(100.0)	133	(88.1)
Mothers referred to Tx during ANC or within one month after delivery	10	(100.0)	31	(91.2)	5	(100.0)	8	(88.9)	17	(100.0)	7	(58.3)	7	(100.0)	4	(50.0)	14	(100.0)	32	(91.4)	135	(89.4)
Referal to Tx for HIV+ mother recorded in patient-retained card	2	(20.0)	10	(29.4)	2	(40.0)	4	(44.4)	15	(88.2)	3	(25.0)	3	(42.9)	0	(0.0)	8	(57.1)	33*	(97.1)	80	(53.3)
Clinic always gives PMTCT drugs for delivery elsewhere	8	(80.0)	29	(85.3)	2	(40.0)	3	(33.3)	3	(17.6)	0	(0.0)	3	(42.9)	2	(25.0)	8	(57.1)	**		58	(50.0)
Location of ART in same building/unit	0	(0.0)	2	(5.9)	0	(0.0)	1	(11.1)	7	(41.2)	1	(8.3)	1	(14.3)	1	(12.5)	2	(14.3)	5	(14.3)	20	(13.2)
***In clinics with ART in different building/unit (n = 131):***																						
Check if woman registers for HIV Tx	9	(90.0)	32	(100.0)	5	(100.0)	8	(100.0)	9	(100.0)	11	(100.0)	5	(71.4)	6	(85.7)	12	(100.0)	29	(96.7)	126	(96.2)
Health worker accompanies woman to HIV Tx	8	(80.0)	29	(90.6)	4	(80.0)	7	(87.5)	5	(55.6)	9	(81.8)	3	(42.9)	2	(28.6)	8	(66.7)	0	(0.0)	75	(57.3)
***Medical management*** (***n*****(%))**																						
Option B+ (Women initiate life-long ART)	2	(20.0)	2	(5.9)	4	(80.0)	1	(11.1)	15	(88.2)	5	(41.7)	4	(57.1)	7	(87.5)	13	(92.9)	10	(28.6)	63	(41.7)

*At least one site with missing data, **>10% of sites with missing data; denominators may vary for categorical variables. ^†^ARV prophylaxis or treatment for mother and prophylaxis for baby. ^‡^Stock-outs ≤1 time in past year, or having no stock outs lasting for two weeks or more.

Regarding PMTCT coordination and integration, anti-retroviral prophylaxis for PMTCT was given on the same day as ANC services in most facilities (88%), and HIV-positive mothers were commonly referred for ART within one month of delivery (89%). Only half (53%) recorded referral in patient-retained cards, and fewer gave PMTCT drugs in advance for home or elsewhere delivery (50%). In facilities where ART was provided in a different building or unit (*n* = 131), most sites checked the woman’s arrival for treatment (96%). There was wide inter-site variation in referrals accompanied by health workers (91% in Kisumu versus none in Manicaland).

The “Option B+” regimen (commencement of life-long treatment during pregnancy) was offered in 42% of facilities, but most common in Karonga (80%), uMkhanyakude (88%), Kyamulibwa (88%) and Rakai (93%).

### Access to HIV treatment

Factors influencing access to HIV treatment were assessed within HIV care units (*n* = 147) (Table 5 and Figure 4c). Although some facilities in Kyamulibwa (40%) and Manicaland (13%) charged some kind of fee (e.g. admission), ART was free in most facilities. ART initiation was available everywhere except Manicaland (61% only provided refills). Nurse-led ART initiation was widespread (78%), except in Nairobi and Kisesa (50%).

**Table 5.** Influences on access to HIV treatment within ART units

	Kenya				Malawi		South Africa				Tanzania				Uganda				Zimbabwe			
	Nairobi		Kisumu		Karonga		Agincourt		uMkhanya-kude		Ifakara		Kisesa		Kyamulibwa		Rakai		Manicaland		Total	(%)
**Total no. clinics offering ART** (***n*(%))**	**8**	**(100.0)**	**32**	**(100.0)**	**5**	**(100.0)**	**9**	**(100.0)**	**17**	**(100.0)**	**12**	**(100.0)**	**4**	**(100.0)**	**10**	**(100.0)**	**14**	**(100.0)**	**15**	**(100.0)**	**126**	**(100.0)**
**Total no. clinics with HIV care (incl. pre-ART)**	**8**	**(80.0)**	**32**	**(100.0)**	**5**	**(100.0)**	**9**	**(100.0)**	**17**	**(100.0)**	**12**	**(100.0)**	**4**	**(100.0)**	**10**	**(100.0)**	**14**	**(100.0)**	**36**	**(100.0)**	**147**	**(100.0)**
***Service access******andcoverage (n(%)) (in clinics with ART)***																						
Free ART	8	(100.0)	32	(100.0)	5	(100.0)	9	(100.0)	17	(100.0)	12	(100.0)	4	(100.0)	6	(60.0)	14	(100.0)	13	(86.7)	120	(95.2)
ART initiation available (in ART clinics)	8	(100.0)	32	(100.0)	5	(100.0)	9	(100.0)	16	(94.1)	12	(100.0)	4	(100.0)	10	(100.0)	14	(100.0)	14	(38.9)	124	(84.4)
Nurses/midwives initiate ART	4	(50.0)	26	(81.3)	5	(100.0)	9	(100.0)	15	(93.8)	8	(66.7)	2	(50.0)	7	(70.0)	11	(78.6)	10	(71.4)	97	(78.2)
**Coor*****dination of care and patient tracking (n(%))***																						
Pre-ART services available	8	(100.0)	31	(96.9)	5	(100.0)	9	(100.0)	16	(94.1)	11	(91.7)	4	(100.0)	10	(100.0)	14	(100.0)	35	(97.2)	143	(97.3)
Pre-ART visit recorded in patient-retained card	4	(50.0)	20	(62.5)	2	(40.0)	3	(33.3)	13	(76.5)	10	(83.3)	4	(100.0)	6	(60.0)	12	(85.7)	31	(86.1)	105	(71.4)
Pre-ART visit recorded on paper or computer at clinic	8	(100.0)	32	(100.0)	5	(100.0)	9	(100.0)	17	(100.0)	11	(91.7)	4	(100.0)	10	(100.0)	13	(92.9)	36	(100.0)	145	(98.6)
Pts. return at least every six months for pre-ART check-up	8	(100.0)	32	(100.0)	5	(100.0)	9	(100.0)	16	(94.1)	12	(100.0)	4	(100.0)	9	(90.0)	14	(100.0)	34	(94.4)	143	(97.3)
***Medical management (n******(%))***																						
*In clinics with any HIV care (pre-ART or ART):*																						
CTX prophylaxis available and in stock in pre-ART	8	(100.0)	32	(100.0)	5	(100.0)	9	(100.0)	15	(88.2)	6	(50.0)	3	(75.0)	10	(100.0)	14	(100.0)	33	(91.7)	135	(91.8)
*In clinics with ART:*																						
TB+ start ART on same day or within two weeks	0	(0.0)	3	(9.4)	4	(80.0)	0	(0.0)	0	(0.0)	7	(58.3)	0	(0.0)	2	(20.0)	5	(35.7)	3	(20.0)	24	(19.0)
ART eligibility with clinical staging only	1	(12.5)	0	(0.0)	1	(20.0)	0	(0.0)	1	(5.9)	0	(0.0)	0	(0.0)	0	(0.0)	0	(0.0)	2*	(13.3)	5	(4.0)
ART eligibility CD4 < 500	0	(0.0)	0	(0.0)	0	(0.0)	0	(0.0)	7	(41.2)	0	(0.0)	0	(0.0)	0	(0.0)	0	(0.0)	0*	(0.0)	7	(5.6)
ART eligibility CD4 ≤ 350	7	(87.5)	31	(96.9)	4	(80.0)	2	(22.2)	9	(52.9)	5	(41.7)	3	(75.0)	9	(90.0)	13	(92.9)	11*	(73.3)	94	(74.6)
Lab tests not required for ART initiation^†^	1	(12.5)	10	(31.3)	5	(100.0)	0	(0.0)	0	(0.0)	4	(33.3)	0	(0.0)	1	(10.0)	0	(0.0)	4	(26.7)	25	(19.8)
No visits required before ART initiation	0	(0.0)	0	(0.0)	0	(0.0)	2	(22.2)	1	(5.9)	0	(0.0)	0	(0.0)	2	(20.0)	3*	(23.1)	0*	(0.0)	8	(6.5)
WHO 2010 first-line ART as standard	3	(37.5)	14*	(43.8)	0	(0.0)	0	(0.0)	1	(5.9)	11	(91.7)	3	(75.0)	7	(70.0)	3	(21.4)	0	(0.0)	42	(33.3)
WHO 2013 first-line ART as standard	4	(50.0)	2*	(6.3)	5	(100.0)	8	(88.9)	16	(94.1)	1	(8.3)	1	(25.0)	3	(30.0)	11	(78.6)	4	(26.7)	55	(43.7)
***Support to PLHIV (n******(%))***																						
No compulsory adherence counselling	0	(0.0)	0	(0.0)	0	(0.0)	1**	(11.1)	7*	(41.2)	0	(0.0)	0	(0.0)	0	(0.0)	1*	(7.1)	0*	(0.0)	9	(7.1)

*At least one site with missing data, **>10% of sites with missing data; denominators may vary for categorical variables. ^†^Liver/renal function and full blood count, excludes CD4.

Only 71% of clinics recorded pre-ART visits on patient-retained cards, with low proportions in Nairobi (50%), Karonga (40%) and Agincourt (33%). Co-trimoxazole prophylaxis (CTX) was in stock in only 50% Ifakara’s facilities and 75% of Kisesa’s, but otherwise available. There were differences in ART initiation among TB-infected PLHIV, with no facilities allowing rapid initiation (same day/within two weeks) in Nairobi, Agincourt, uMkhanyakude and Kisesa, vs. 80% in Karonga. Few facilities conducted ART eligibility assessment with clinical staging only. Most sites initiated treatment with a CD4 count of ≤350 cells/mm^3^, except uMkhanyakude where 41% initiated ≤500 cells/mm3. Some still initiated at ≤250 cells/mm3 (78% Agincourt, 58% Ifakara and one facility in each of Kisumu, Kisesa, Masaka and Rakai) (data not shown). Only 20% facilities required no additional laboratory tests before treatment initiation, except Malawi where none required them. A few clinics in Agincourt, Kyamulibwa and Rakai allowed patients to initiate at first contact with the clinic. More facilities (44%) used WHO’s 2013 first line ART regimen (containing tenofovir), compared to 33% using WHO’s 2010 regimen, and this was most common in Malawi and South Africa.

Adherence counselling was compulsory everywhere except one clinic in Agincourt and Rakai, and seven in uMkhanyakude.

### Retention on HIV treatment

Factors influencing retention were assessed within facilities providing ART (*n* = 126) (Table 6 and Figure 4d). National treatment guidelines were mostly available, though less so in Karonga (60%) and Manicaland (40%). Most staff underwent recent ART training, though less commonly in Ifakara (25%) and Manicaland (40%). Well-stocked facilities were common, but infrequent in Tanzania: 8.3% in Ifakara, none in Kisesa with opportunistic infection drugs; 75% in Ifakara, 50% in Kisesa with ART drugs.

**Table 6.** Influences on retention on ART

	Kenya				Malawi		South Africa				Tanzania				Uganda				Zimbabwe			
	Nairobi		Kisumu		Karonga		Agincourt		uMkhanya-kude		Ifakara		Kisesa		Kyamulibwa		Rakai		Manicaland		Total	(%)
**Total no. clinics offering ART (*****n*****(%))**	**8**	**(100.0)**	**32**	**(100.0)**	**5**	**(100.0)**	**9**	**(100.0)**	**17**	**(100.0)**	**12**	**(100.0)**	**4**	**(100.0)**	**10**	**(100.0)**	**14**	**(100.0)**	**15**	**(100.0)**	**126**	**(100.0)**
***Quality of care (n******(%))***																						
Treatment guidelines available^†^	8	(100.0)	27	(84.4)	3	(60.0)	9	(100.0)	17	(100.0)	12	(100.0)	4	(100.0)	7	(70.0)	12	(85.7)	6	(40.0)	105	(83.3)
≥1 staff trained on ART in past two yrs	8	(100.0)	32	(100.0)	4	(80.0)	8	(88.9)	13	(76.5)	3	(25.0)	4	(100.0)	9	(90.0)	14	(100.0)	6	(40.0)	101	(80.2)
QOC audits at least once a year in ART	7	(87.5)	31	(96.9)	4	(80.0)	6	(66.7)	17	(100.0)	12	(100.0)	3	(75.0)	8	(80.0)	14	(100.0)	15	(100.0)	117	(92.9)
OI prophylaxis well-stocked^‡^	6	(75.0)	26	(81.3)	4	(80.0)	8	(88.9)	13	(76.5)	1	(8.3)	0	(0.0)	7	(70.0)	12	(85.7)	13	(86.7)	90	(71.4)
First-line ARVs well-stocked^‡^	8	(100.0)	30	(93.8)	4	(80.0)	9	(100.0)	15	(88.2)	9	(75.0)	2	(50.0)	8	(80.0)	12	(85.7)	14	(93.3)	111	(88.1)
***Coordination of care and patient tracking (n(%))***																						
Six-monthly CD4 monitoring (min)	6*	(85.7)	31	(96.9)	2	(40.0)	1	(11.1)	2	(11.8)	12	(100.0)	4	(100.0)	9	(90.0)	2	(14.3)	15	(100.0)	84	(67.2)
Three-monthly drug supplies given	5	(62.5)	25	(78.1)	0	(0.0)	3	(33.3)	0	(0.0)	1	(8.3)	1	(25.0)	2	(20.0)	14	(100.0)	1	(6.7)	52	(41.3)
Drugs collectable by designee	7	(87.5)	32	(100.0)	5	(100.0)	6	(66.7)	16	(94.1)	12	(100.0)	2	(50.0)	10	(100.0)	14	(100.0)	15	(100.0)	119	(94.4)
Pill counts at every visit	5	(62.5)	25	(78.1)	5	(100.0)	8	(88.9)	6	(35.3)	7	(58.3)	4	(100.0)	8	(80.0)	11	(78.6)	13*	(92.9)	92	(73.6)
Home visits following poor adherence	1	(12.5)	11	(34.4)	0	(0.0)	1	(11.1)	0	(0.0)	0	(0.0)	0	(0.0)	2	(20.0)	8	(57.1)	4	(26.7)	27	(21.4)
Home/phone contact after missed visit	8	(100.0)	31	(96.9)	4	(80.0)	9	(100.0)	17	(100.0)	5	(41.7)	4	(100.0)	10	(100.0)	13	(92.9)	12	(80.0)	113	(89.7)
LTFU defined as 90 days^§^	5**	(71.4)	28*	(90.3)	1	(20.0)	9	(100.0)	17	(100.0)	11*	(100.0)	3**	(100.0)	8*	(88.9)	14	(100.0)	12**	(92.3)	108	(90.8)
TB treatment available in facility	8	(100.0)	32	(100.0)	3	(60.0)	9	(100.0)	17	(100.0)	11	(91.7)	4	(100.0)	10	(100.0)	9*	(69.2)	15	(100.0)	118	(94.4)
***Support to PLHIV (n*******(%))****																						
≥1 adherence session required	8	(100.0)	32	(100.0)	5	(100.0)	6**	(85.7)	9*	(56.3)	12	(100.0)	4	(100.0)	10	(100.0)	12*	(92.3)	13**	(100.0)	111	(92.5)
Individual counselling available	6	(75.0)	1	(3.1)	5	(100.0)	8**	(100.0)	16	(94.1)	12	(100.0)	1	(25.0)	8	(80.0)	13	(92.9)	10**	(76.9)	80	(65.0)
Support groups available^¶^	8	(100.0)	32	(100.0)	5	(100.0)	7	(77.8)	8	(47.1)	9	(75.0)	4	(100.0)	5	(50.0)	13	(92.9)	13	(86.7)	104	(82.5)
Nutritional supplements for malnourished available^¶^	8	(100.0)	32	(100.0)	5	(100.0)	9	(100.0)	17	(100.0)	1	(8.3)	2	(50.0)	5	(50.0)	7	(50.0)	12	(80.0)	98	(77.8)
Home-based care available^¶^	7	(87.5)	30	(93.8)	5	(100.0)	9	(100.0)	16	(94.1)	12	(100.0)	4	(100.0)	7	(70.0)	13	(92.9)	15	(100.0)	118	(93.7)
***Medical management (n(%))***																						
Prophylactic IPT offered and in stock	5	(62.5)	19	(59.4)	5	(100.0)	9	(100.0)	14	(82.4)	0	(0.0)	2	(50.0)	0	(0.0)	0	(0.0)	0	(0.0)	54	(42.9)
TB screening at every ART visit	8	(100.0)	32	(100.0)	4	(80.0)	9	(100.0)	17	(100.0)	9	(75.0)	4	(100.0)	9	(90.0)	13	(92.9)	13	(86.7)	118	(93.7)

*At least one site with missing data, **>10% of sites with missing data; denominators may vary for categorical variables.

^†^National guidelines, seen or not seen, any guideline. ^‡^Stock-outs ≤1 time in past year, or having no stock outs lasting for two weeks or more; OI drugs are co-trimoxazole, fluconazole or IPT. ^§^Loss to follow-up defined as no contact within 90 days of last scheduled visit. ^¶^Onsite or through referral within district.

Two-thirds of facilities conducted at least six-monthly CD4 count monitoring, but this was low in Agincourt (11%), uMkhanyakude (12%) and Rakai (14%). About 41% of facilities gave three-monthly supplies of drugs but 94% allowed drug collection by a designee. Pill counts at every visit were common (74%) but infrequent in uMkhanyakude (35%). While few sites (21%) conducted home visits following poor adherence, most did so after a missed appointment (90%), although not in Ifakara (42%).

Nearly all facilities required more than one adherence counselling session (93%), but not in uMhkanyakude (56%). Many (65%) offered individual counselling but this was low in Kisumu (3%) and Kisesa (25%). Support groups were generally available, although offered by only half of facilities in uMkhanyakude and Kyamulibwa.

Prophylactic Isoniazid Preventive Therapy (IPT) for TB prevention was offered and in-stock in under half of facilities, and not available anywhere in Ifakara, Kyamulibwa, Rakai or Manicaland. TB screening was conducted at every visit in most facilities (94%).

## Discussion

This study provides a detailed picture of HIV service delivery in 156 facilities in six sub-Saharan countries, highlighting substantial variation within and between countries in programme-level indicators influencing HIV service uptake and retention in care. Comparative multi-country surveys of HIV service delivery quality have been limited, but have also shown substantial variation between settings [12]. And while a recent meta-analysis and review have both demonstrated the critical role that health services play in influencing retention in care [13,14], to our knowledge this is the first study across the Eastern and Southern African region comparing facility performance over the whole continuum of HIV care, including influences on testing uptake, initiation of, and retention on ART. The assessment of service performance using a standardized instrument across the 10 sites is useful both for programme monitoring and for benchmarking performance between settings and over time. The HDSS that form the ALPHA Network provide important demographic parameters for national policy-makers [2,15], and this survey provides critical contextual information to help explain differences in HIV service access by the local populations, and ultimately differences in HIV-related mortality through the HIV treatment cascade.

Many of the facilities we surveyed performed well across multiple indicators that may be expected to impact positively on service utilization. The near-universality of free HIV services, high levels of PMTCT provision within ANC, pre-ART monitoring availability and adherence counselling were impressive, demonstrating the remarkable progress made in the provision of HIV care in the region since the commencement of ART rollout. However, several areas were identified with inconsistency in service provision across the continuum of care. Differences within or between sites and countries indicate areas of opportunity for increasing patient engagement.

There were common service gaps influencing HIV testing access. The high testing volume in some facilities was worrying, as well as frequently reported test kit stock-outs. Poor quality testing may undermine uptake and/or the feasibility of annual testing, recommended by WHO [16]. Furthermore, a growing body of evidence indicates the importance of community-based testing approaches, including mobile outreach [13,17,18], approaches often absent in facilities surveyed. High-risk groups were only targeted in Nairobi, suggesting an “invisibility” in the other predominantly rural locations surveyed. It was also particularly alarming that, with the exception of South Africa, pre- and post-testing counselling services were not consistently provided anywhere. This has implications on ascertainment of consent, patient understanding, provision of psychosocial support and linkage to care.

Findings also indicated weaknesses in service coordination and patient tracking, which may be particularly influential on high rates of attrition across the HIV cascade documented in multiple sub-Saharan settings [6,8,9]. Effective referral must be strengthened if linkage to HIV treatment is to be improved.

The starkest differences between sites were in factors likely to influence access to ART, with Malawian facilities performing particularly well across a range of indicators in this area, including nurse-led ART initiation, ART initiation without laboratory testing and PITC for pregnant women with PMTCT Option B+ for those testing positive. Malawi often stands out for its progressive HIV policies [10], despite being one of the poorest African countries, and our findings suggest that policies are translating into practice, even in rural areas. Other sites, notably those in Kenya, performed less well across the same indicators, despite having a substantially higher per capita HIV budget and health worker ratios [19]. The impact of rapid initiation policies on long-term adherence, however, remains under-studied.

All sites exceled in some areas, but lagged in others. For example, many facilities in Tanzanian sites reported regular drug or supply stock-outs, likely to impact negatively on patient progression and clinical outcomes, but generally performed well in supporting PLHIV on ART, likely impacting positively on retention and adherence. Multiple instances of variation within countries were observed where two sites were studied (Uganda, Kenya, South Africa, Tanzania) suggesting that national HIV policy differences only partly explain variation in service provision. For example, in Kenya, nurse-led ART initiation was very common in Kisumu but implemented only in half of Nairobi facilities, despite national policy [10]. In Tanzania, rapid ART initiation for TB+ was allowed in most facilities in Ifakara, but none in Kisesa.

The causes of variation in service performance between sites and across countries are likely to be multi-faceted, and may include national policy variation (although some policies common to all six countries, such as repeat HIV testing in pregnancy, or pill counts at every visit, were not always implemented in practice), the complexity of the policy and its ease of implementation, the programmatic support to ensure providers are trained and practicing it, and a wide range of structural factors influencing quality of care, such as provider remuneration and motivation, training, drugs/supplies procurement processes, and supervision and management [20,21]. Further research is planned by the ALPHA Network to investigate the extent to which the observed service delivery reflects national policy differences; the local factors influencing policy implementation; and the association between policy implementation, cascade progression and mortality. Research is also being undertaken within the HDSS to explore health-seeking behaviour, to understand the normative and social drivers of service utilization across the continuum of HIV care.

### Limitations

The survey was designed to be representative of HDSS service provision, thus national representation should not be implied. However, most facilities surveyed are considered typical of national service provision, except three large facilities in Rakai, Masaka and Kisumu. These sites received additional support and thus quality observed may have been higher than average. However, there may be differences in treatment-seeking behaviour in these HDSS due to regular population-based HIV testing, leading to higher volumes of patients than might otherwise be expected.

The completion of the survey by facility managers presents a potential reporting bias, and results may not reflect the reality of care quality received by patients. The study dates (with fieldwork conducted in 2013/2014) should also be considered in interpreting findings; important international policies such as Option B+ had only been recommended by WHO in June 2013, and were not expected to be widely implemented at the time of the study. Lastly, while the survey design and conceptual framework were guided by a literature review and aimed to be comprehensive, certain indicators impacting on access to HIV care may have been missed, in part due to the rapidly evolving field. Future research should consider capturing the ART monitoring strategy used (e.g. whether routine viral load monitoring offered; or availability of point-of-care CD4 testing); partner testing in ANC; the number of pre-ART counselling sessions required; or actions taken when PLHIV do not register in care post-referral.

## Conclusions

This study identified an overall high standard of HIV service delivery performance across six countries in sub-Saharan Africa, but with substantial variability in service indicators expected to impact on uptake of the continuum of HIV care. Inter- and intra-country differences represent opportunities to improve the delivery of comprehensive services to PLHIV. Patient engagement across this continuum is likely to remain sub-optimal unless issues relating to service access, quality of care and coordination are consistently improved in practice. Policy-makers must act on the weaknesses identified, in particular poor performance on testing accessibility, as well as deficiencies in coordination of care and patient tracking. Such action will be essential to support implementation of WHO’s new “test and treat” strategy [10], since success is contingent upon high levels of HIV testing, linkage to care and retention following initiation.
